# University Students’ Motives-for-Physical-Activity Profiles: Why They Practise and What They Get in Terms of Psychological Need Satisfaction

**DOI:** 10.3389/fpsyg.2020.621065

**Published:** 2021-01-12

**Authors:** Rafael Valenzuela, Nuria Codina, José Vicente Pestana

**Affiliations:** Department of Social Psychology and Quantitative Psychology, University of Barcelona, Barcelona, Spain

**Keywords:** physical activity, psychological need satisfaction, motives, cluster analysis, self-determination theory

## Abstract

Physical activity (PA) is an important habit for overall health and quality of life, but it tends to recede as young adults transition from high school into university. The present study sought to understand, in the case of university students that still practice PA, their motives for PA and their relationships with psychological need satisfaction (PNS) and characteristics of practice regularity (frequency, duration, team, competitive, coach, league, federation, and type of day of the week for PA). Participants were 423 university students who reported to practice PA (203 identified as men, 191 as women, 29 did not report gender), with ages ranging from 18 to 30 years old (*M* = 19.91, *SD* = 1.97). Measures assessing motives for PA, PNS, and PA characteristics were completed. Hierarchical, followed by iterative, cluster analysis was used and four naturally occurring groupings of university students were identified based on their motives for PA: one *extrinsic-motives* cluster (with both extrinsic motives—fitness and appearance—above the mean), one *all-motives* cluster (with all five motives above the mean), one *intrinsic-motives* cluster (with all three intrinsic motives—enjoyment, competence, social—above the mean), and one *low-motives* cluster (with all motives below the mean). Groupings were compared in terms of the characteristics of their practice regularity (frequency, duration, competition, team, coach, league, federation, type of day of the week used for PA) and their levels of PNS (of the needs for autonomy, competence, and relatedness in PA). Significant between-group differences were observed in the duration of single principal PA sessions, minutes per week practicing main PA, total PA minutes per week, and type of day of the week used for PA. The number of days per week devoted to the principal PA and the number of total PAs practiced were similar across all four clusters. With regard to between-group differences in psychological need satisfaction in PA by cluster, these analyses showed the existence of four clearly distinguishable naturally occurring groupings based on motives for PA, which gives researchers and practitioners the possibility to analyze and implement tailored interventions aimed at promoting PA among university students.

## Introduction

Physical activity (PA) is important in preventing severe health risks (Krishnan et al., 2009; Heath et al., 2012; Piercy et al., 2018) as well as in yielding positive health benefits derived from its practice (Kim et al., 2016; Pacesova et al., 2019). However, regularity of practice has proven to be a critical aspect, closely linked with both aforementioned types of favorable consequences of PA. Specifically, the World Health Organization (WHO) and the European Commission recommend that adults practice moderate PA a minimum of 150 min per week, vigorous PA 75 min/week, or a combination of the two (World Health Organization, 2010; Powell et al., 2011). Yet, these standards are far from being met among the general population, as more than two-thirds of Europeans practice little or no PA (European Commission, 2018), and in the case of the Spanish population, 34.4% do not engage in any PA, 38.9% practice insufficient PA, and the remaining 26.7% practice enough PA, but mostly of moderate intensity (Ministerio de Educación, Cultura y Deporte, 2016).

As a matter of fact, it has been reported that PA tends to recede in young adulthood and in the transition into university studies (Maselli et al., 2018). At the same time, a worrisome decline in health-favorable activities such as physical education (Marshall and Hardman, 2000) and outdoor play time in children (Clements, 2004) strongly contrasts with increments in online entertainment (Chahal et al., 2013). In this regard, it has been argued that PA requires promotion in schools, through facilitation of intrinsic motivation (i.e., sports that are liked by students), teacher support, development of students’ perceived competence, and enhancement of PA values, through the provision of information about health benefits (Ntoumanis, 2005; Codina et al., 2016).

On the one hand, reasons for concern are that higher education itself has been linked with lesser PA and that higher education institutions, in the European and Spanish contexts, scarcely promote its practice among their students (Moreno-Arrebola et al., 2018; Mella-Norambuena et al., 2019). On the other hand, being hopeful is possible, given that higher education is also a time in which healthy habits and lifestyles can be built up (Maselli et al., 2018). For these reasons, it is important to understand both what personal variables drive university students to practice PA (Gunnell et al., 2014; Ingledew et al., 2014; Lindwall et al., 2016; Caro-Freile and Rebolledo-Cobos, 2017) and what contextual variables are associated with this practice, for instance, support from others (e.g., team or family members), or the organization and time frame linked to the activity (Codina and Pestana, 2012; Codina et al., 2020).

### Motives for Physical Activity and Their Relationships With Goals and Psychological Needs

Motives have been defined as internal processes that give energy and direction to behavior and may originate in real needs, but also in cognitions or emotions, which may be aligned with these needs or not (Reeve, 2009), such as those present in personal expectations or plans. Thus, needs, cognitions, and emotions share a common ground as three specific types of internal motives, which answer the question *why* do I want (to do) that.

Following the previous reasoning, according to the self-determination theory (SDT), motives can serve the satisfaction of basic psychological needs, such as autonomy (feeling as the cause of one’s actions), competence (perceiving oneself as capable of accomplishing specific goals), and relatedness (perceiving oneself as not emotionally isolated from others), which are key nutriments for the development of quality motivation and personal growth (Deci and Ryan, 2000; Ryan and Deci, 2017). But, additionally, motives can also serve cognitions and emotions included in personal goals. Goals have been defined as cognitive representations of desired endpoints that have effects on the individuals’ evaluations, emotions, and behaviors; compete against each other; fluctuate in accessibility; and have a complex nature, as they are built on multiple integrated memories (Fishbach and Ferguson, 2007). Goals have structure and contents, vary in abstractness, and represent memory-based desired end states, which the individual is likely to pursue with increasing drive, until it is reached or the costs of pursuit become excessive (Fishbach & Ferguson, ibidem). As regards their value, goals can have both intrinsic and extrinsic goal contents (Sebire et al., 2008); thus, it has been argued that all goals are not created equal (Ryan et al., 1996) and that distinctions are required between different types of goals and their contents, as these distinctions are relevant for understanding the nature of derived motives.

Motives for physical activity have been theorized (Frederick and Ryan, 1993) considering the differentiation between intrinsic motives—obtained or accomplished in the course of practice (Deci, 1975)—and extrinsic motives—obtained as practice-derived results (Deci and Ryan, 2000), and also distinguishing between intrinsic and extrinsic goal contents in exercise, respectively, reflecting an intrinsic or extrinsic value of the desired endpoint (e.g., losing weight, respectively, for health or appearance purposes: Sebire et al., 2008).

Derived from these theoretical underpinnings, an early three-factor measure of motivation for physical activity within SDT (Motivation for Physical Activities Measure—MPAM; Frederick and Ryan, 1993) included enjoyment/interest, skill development, and body-related motives. The enjoyment/interest motive clearly represents an intrinsic motive, as it is satisfied within practice itself, thus, serving intrinsic motivation (which is very much a synonym of enjoyment and interest). Furthermore, this motive also serves the satisfaction of both basic psychological needs for autonomy and competence (Ryan and Deci, 2000), given that people who practice intrinsically motivating activities value both experiencing enjoyment and interest, but also feeling competent in practice (Deci, 1975). The skill development motive also represents an intrinsic motive, as it is also satisfied within practice itself, when the individuals’ performance holds up to the expectations and the level of the challenges faced. Additionally, this motive serves the basic psychological need for competence, by supporting growth and development. Lastly, the body-related motive was theorized as extrinsic, given that, based on this motive, engagement in practice is instrumental in achieving a goal that is an indirect result derived from the activity, and because the notion of body-related was mostly associated with appearance.

Building on these early theoretical and measurement approaches, further developments (Motives for Physical Activity Measure – Revised—MPAM-R: Ryan et al., 1997) included enjoyment and competence motives and additionally a social motive, in this way acknowledging that PA could be prompted by the pursuit of satisfaction of the basic psychological need for relatedness or social connectedness. Furthermore, this revised approach took into account that the goal contents of a so-called body-related motive could have both an intrinsic (building health) and an extrinsic value (looking good to others). Thus, the MPAM-R “split” the body-related motive into two factors: respectively, fitness/health and appearance motives.

Consequently, research using this measure can distinguish between intrinsic and extrinsic motives and between motives with intrinsic and extrinsic goal contents. Both the enjoyment and competence motives can be considered intrinsic and are clearly related with intrinsic motivation and with psychological need satisfaction (PNS) of the needs for autonomy (e.g., choosing freely how to participate in enjoyable dance lessons) and competence (e.g., accomplishing an intended time mark while running a marathon, or a precise movement while dancing). The social motive can also be considered intrinsic and related with the satisfaction of the basic psychological need for relatedness (e.g., feeling a sense of emotional connectedness with teammates while playing volleyball, basketball, soccer, or rugby). Lastly, the MPAM-R can also distinguish between two extrinsic motives, such as fitness/health and appearance, which coincide in pursuing PA for its indirect results, but diverge in the value (respectively, intrinsic vs. extrinsic) of the goal contents, pursuing, respectively, health-related outcomes versus outcomes linked to social recognition, both based on cognitions or emotions.

Research with the Spanish version of the MPAM-R found that women scored higher than men both in enjoyment and in appearance motives; young people aged between 16 and 24 years old scored higher in the appearance motive than older age groups, and older participants, in turn, scored higher in the fitness motive relative to younger groups; furthermore, people practicing more than 3 days per week scored in average higher in all five motives than people practicing less; people practicing with coach or direction reported higher enjoyment, competence, and fitness motives than those practicing on their own; and lastly, people practicing with friends reported a higher social motive than those practicing alone (Moreno-Murcia et al., 2007).

Furthermore, research with the MPAM-R in active women has shown that motives are associated with psychological need satisfaction, except for the appearance motive (Wilson et al., 2002). Moreover, all five motives have been positively associated with participation in PA, but the three intrinsic motives (enjoyment, competence, and social) have shown the most robust associations with autonomous forms of motivation, whereas appearance and fitness motives have shown to be associated with extrinsic autonomous but also extrinsic controlled (introjected) types of motivation (Battistelli et al., 2016).

PNS, for instance, in school physical education, is supposed to originate quality motivation, which in turn enhances several positive outcomes like affect and intentions to participate (Ntoumanis, 2005). Furthermore, competence need satisfaction and relatedness need satisfaction have been shown to interact, respectively, with achievement and affiliation motives in predicting outcomes like flow in sport (Schüler and Brandstätter, 2013), suggesting that specific motive profiles may benefit most from satisfaction of psychological needs that best produce favorable outcomes at a well-being level through their interactions with already preferred motives.

Interestingly, previous (longitudinal) research on PNS in exercise has found that—somewhat contrastingly with SDT assumptions—adherence to exercise program was associated with reduced perceptions of autonomy and, furthermore, that relatedness was not associated with autonomy nor autonomous regulation (Wilson et al., 2003). This suggests exploring the role of relatedness as a potential promoter of motive internalization, leading to less autonomous regulation. Also, autonomy need satisfaction, in particular, has been linked with the development of behavioral regulation based on self-concordant cognitions and emotions (i.e., identified regulation; Ryan and Deci, 2000), which in turn is consistent with persistence in exercise and well-being (Wilson et al., ibidem). This, in turn, suggests analyzing the ways in which the endorsement of distinct motivation bases for exercise may be associated with distinct adherence behaviors and aspects of psychological well-being (which has been underlined, among others, by Gómez-Mazorra et al., 2020).

Lastly, approaches based on SDT, especially on psychological need satisfaction, have shown favorable effects in the implementation of interventions aimed at increasing PA practice among the youth (Kim et al., 2015; Muftuler and Ince, 2015; Chacón-Cuberos et al., 2019; Corella et al., 2019). In general, these findings suggest the importance of in-depth analysis of the *whys* of PA as indispensable elements for future intervention programs aimed at increasing PA among university students.

### The Present Study

The conceptual and practical overview presented above shows that internal motives are reasons people have for doing things, which may be based on needs, cognitions, or emotions (Reeve, 2009). In the case of motives for PA, it is important to understand if these could predict behaviors of PA practitioners and the outcomes or benefits derived from the activity.

In this regard, it has been noted that it is most relevant to address the question if behavior is intrinsically motivated (Deci, 1975): this is a self-satisfying end in itself; or, alternatively, if behavior is extrinsically motivated, this is an indirect means to an end or result derived from the activity; or if behavior is the result of a combination of both intrinsic and extrinsic motivation. Furthermore, in line with the goal contents theory (GCT, a mini theory within SDT: Vansteenkiste et al., 2010), it is also relevant to address the question if the contents of each goal have intrinsic or extrinsic value (Sebire et al., 2008).

Antecedents of cluster analysis using the Spanish MPAM-R (Nuviala Nuviala et al., 2013) have reported the emergence of three groupings: a first one with above-average scores in all motives; a second one with average fitness and appearance motives and below-average intrinsic motives; and a third one with above-average enjoyment and social motives, an average competence motive, and below-average extrinsic motives.

By and large, SDT argues that PNS promotes quality motivation (including intrinsic motives) and, contrarily, low levels of PNS or unfulfilled intrinsic motives may foster extrinsic motives, which then may act as compensations for low PNS (Ryan and Deci, 2000).

Based on the aforementioned antecedents, the present study focused on three objectives:

• Identifying distinct natural groupings based on motives for PA among university students (comparable with a previous work with the MPAM-R).
• Analyzing if potentially emerging clusters would diverge in their characteristics of PA practice regularity.
• Assessing if potentially emerging clusters would diverge in their levels of psychological need satisfaction in PA.

In a more general sense, these three objectives allow for the discussion of naturally occurring groupings with distinct profiles of motives for PA, based on different combinations of intrinsic and extrinsic motives and goal contents. The study also allows for the discussion of associations between profiles of motives for PA and a) various characteristics of PA practice regularity and b) diverse levels of psychological need satisfaction in PA. In this regard, the present study contributes knowledge about motivational dynamics in physical activity aimed at improving the understanding, promotion, and management of PA participation.

## Materials and Methods

### Design

In order to evaluate the research objectives, a cross-sectional non-experimental predictive design was carried out according to the taxonomy proposed by Ato et al. (2013).

### Participants

A convenience sample of 423 university students who reported practicing PA participated in the present study. Among the participants, 203 identified themselves as men and 191 as women, and 29 did not report gender. Their ages ranged from 18 to 30 years old; *M* = 19.91, *SD* = 1.97. In order to study PA motive profiles in youth and young adults, 10 students over the age of 30 years old were excluded from the analyses. Participation in the study was voluntary (i.e., participants did not receive any payment or academic benefit, such as extra marks in study subjects or course credit).

### Measures

Data was collected using three instruments, accompanied by the required demographics.

*Motives for physical activity* were assessed with the Spanish version (Moreno-Murcia et al., 2007) of the MPAM-R (Ryan et al., 1997). Cronbach’s alphas for the original English (and Spanish) language versions were as follows: α_enjoyment_ = 0.92 (0.84), α_competence_ = 0.91 (0.85), α_social_ = 0.83 (0.81), α_fitness_ = 0.78 (0.80), and α_appearance_ = 0.88 (0.87), whereas in the present study, alphas were, respectively, 0.91, 0.86, 0.87, 0.82, and 0.89.

*Psychological need satisfaction* was assessed with the Spanish version (Moreno-Murcia et al., 2011) of the Psychological Need Satisfaction in Exercise Scale (Wilson et al., 2006). Cronbach’s alphas for original English (and Spanish) language versions were as follows: α_competence_ = 0.91 (0.80), α_autonomy_ = 0.91 (0.69), and α_*relatedness*_ = 0.90 (0.73), whereas in the present study, alphas were, respectively, 0.89, 0.86, and 0.85.

*Regularity of physical activity* was measured with an *ad hoc* self-report questionnaire assessing one main physical activity and overall weekly time investment in physical activities. The survey comprised time investment in PA, including months per year, frequency, days per week, and minutes per session of the main activity practiced, as well as total number of activities practiced, total minutes per week, and type of day of the week practiced. Furthermore, structure and regularity characteristics of the main physical activity were assessed, such as whether practice happened in a team, competitively, with a coach, or in a league or federation.

### Procedure

Before collecting data, we contacted the academic office of the faculties whose students would take part in the sample. After obtaining the corresponding authorization for the research, regular course teachers allowed researchers to address their students in the final minutes of the class, inviting them to participate in a survey about university students’ uses of time, habits, and experience. The students were allowed to continue participating only if they agreed to sign the informed consent, participating voluntarily, and anonymously accessing questionnaires on Qualtrics platform *via* web links or QR codes.

The ethical requirements of the University of Barcelona Bioethics Commission (CBUB, Institutional Review Board IRB00003099) were applied to the current study, which meant that additional approval for the research was not required because the data obtained did not involve animal or clinical experimentation. Additionally, this study complies with the recommendations of the General Council of Spanish Psychological Associations (Consejo General de Colegios de Psicólogos), the Spanish Organic Law on Data Protection (Jefatura del Estado, 1999), and the Declaration of Helsinki (World Medical Association, 2013).

### Analysis

Means, standard deviations, skewness, kurtosis, and normality are described in Table 1, and bivariate correlations among study variables are presented in Table 2. To assess if distinct groupings of university students occurred based on their motives for physical activity, we conducted hierarchical (Ward’s method) cluster analysis to assess the number of groups, followed by iterative cluster analysis to make groupings more precise. We used final group centers from the hierarchical method as initial centers for the iterative method, iterating until the change between cluster centers was less than 2% of the minimum initial distance between centers. We assessed the stability of hierarchical and iterative solutions *via* Cohen’s Kappa.

**TABLE 1:** Means, standard deviations, skewness, kurtosis, and normality for PA motives, regularity characteristics of practice, and psychological need satisfaction.

	Min–max	*M*	*SD*	Skewness	Kurtosis	*p*
Enjoyment motive	1–7	5.92	1.14	–1.48	2.33	<0.001
Competence motive	1–7	5.61	1.18	–0.95	0.59	<0.001
Social motive	1–7	4.62	1.71	–0.61	–0.54	<0.001
Fitness motive	1–7	5.88	1.08	–1.26	1.87	<0.001
Appearance motive	1–7	4.73	1.54	–0.50	–0.52	<0.001
Months per year PA1	1–12	9.72	2.85	–1.36	0.99	<0.001
Frequency PA1	1–6	5.61	0.91	–2.57	6.69	<0.001
Days per week PA1	1–7	3.28	1.36	0.41	–0.11	<0.001
Minutes per day PA1	15–750	109	76	3.32	17.18	<0.001
Minutes per week PA1	0–3,000	276	304	3.33	20.15	<0.001
Number of PAs total	1–9	1.51	1.09	3.56	16.62	<0.001
Minutes per week PA total	0–3,000	338	382	2.93	13.15	<0.001
Competence need satisfaction	1.50–6.00	4.87	0.83	–0.69	0.92	<0.001
Autonomy need satisfaction	1.33–6.00	4.60	1.03	–0.68	0.03	<0.001
Relatedness need satisfaction	1.00–6.00	4.12	1.19	–0.81	0.35	<0.001

**TABLE 2:** Intercorrelations between PA motives (1–5), characteristics of practice (6–11), and psychological need satisfaction (12–14).

		1	2	3	4	5	6	7	8	9	10	11	12	13	14
1	Enjoyment motive														
2	Competence motive	0.58**													
3	Social motive	0.50**	0.35**												
4	Fitness motive	0.21**	0.50**	0.05											
5	Appearance motive	–0.07	0.23**	–0.09	0.58**										
6	Months per year PA1	0.07	0.14**	–0.04	0.19**	0.13**									
7	Frequency PA1	0.12*	0.18**	–0.04	0.26**	0.19**	0.50**								
8	Days per week PA1	0.10	0.05	0.00	–0.07	–0.04	0.24**	c							
9	Minutes per day PA1	0.13**	0.02	0.25**	−0.24**	−0.28**	−0.17**	−0.30**	0.13*						
10	Minutes per week PA1	0.13**	0.13**	0.13**	–0.03	–0.08	0.26**	0.40**	0.56**	0.49**					
11	Total number of PAs	0.09	0.12*	0.00	0.06	0.03	0.07	–0.04	0.11*	0.04	0.03				
12	Total minutes per week	0.14**	0.12*	0.11*	–0.03	–0.06	0.22**	0.31**	0.53**	0.41**	0.85**	0.31**			
13	Competence NS	0.45**	0.49**	0.22**	0.16**	0.02	0.21**	0.17**	0.19**	0.09	0.19**	0.13**	0.20**		
14	Autonomy NS	0.13**	0.19**	−0.13**	0.22**	0.18**	0.08	–0.04	0.02	0.02	–0.08	0.11*	–0.06	0.33**	
15	Relatedness NS	0.31**	0.24**	0.65**	–0.07	−0.17**	0.07	0.04	–0.01	0.24**	0.15**	–0.01	0.11*	0.31**	–0.06

PA1 = main physical activity in terms of regularity of practice; NS = need satisfaction; *p < 0.05 (two-tailed); **p < 0.01 (two-tailed); c = at least one of the variables is constant (days per week = 0, for less-than-weekly frequency PA1).

## Results

All study variables had non-normal distributions. The motives that received the highest average scores were enjoyment and fitness, followed by competence; in turn, social and appearance motives yielded the lowest average scores and the highest variability (Table 1). Scores for PNS were all robust-to-high, the highest average (with the lowest variability) being competence need satisfaction (Table 1).

Participants practiced between one and nine PAs (*M* = 1.52, *SD* = 1.09). Regarding their main PA (hereinafter, PA1), a vast majority practiced it with a close-to-weekly frequency, in average, 9.67 months per year (*SD* = 2.9), 3.27 days per week (*SD* = 1.35), 109 min per day (*SD* = 75), and 274 min per week (*SD* = 302); total time devoted to main and additional PAs added up to 339 min per week (*SD* = 381) (Table 1). Most students practiced PA only on weekdays (40.9%), a minority practiced only on weekends (14.1%), approximately one in every three practiced both on weekdays and weekends (35.6%), and one in every 10 students practiced PA without a specific set day to do it (9.5%). To some extent, PA was practiced competitively (41%), as a team (52%), with a coach (50%), in a league (28%), and as part of a federation (28%).

As can be observed in Table 2, intrinsic motives (enjoyment, competence, social) showed robust internal consistency; also, fitness and competence motives showed a positive bivariate correlation; and, lastly, both extrinsic motives, appearance and fitness, were also positively related. As regards PNS, competence was positively related with both autonomy and relatedness, but relatedness and autonomy were not associated. As regards the 15 possible associations between the five motives and the three psychological needs, interestingly, all were positive and significant except for four: competence need satisfaction was negatively related with the social motive, and relatedness need satisfaction was negatively associated with the appearance motive; and furthermore, relatedness need satisfaction was not associated with the fitness motive, and autonomy need satisfaction was not correlated with the appearance motive. Contrarily, positive robust associations were found, respectively, between autonomy need satisfaction and enjoyment and competence motives and between relatedness need satisfaction and the social and enjoyment motives.

Table 2 also shows that frequency of practice (at least once a year, semester, trimester, month, fortnight, week) was positively related with all motives except the social motive, the association with the greatest coefficient being that with the fitness motive. Number of days per week was not related with motives, but minutes per day was positively related with the enjoyment and social motives and negatively with extrinsic motives (fitness and appearance). Also, minutes per week of main PA and total minutes per week (all activities) were positively, though weakly, associated with all three intrinsic motives. Minutes per day was negatively associated with months per year and frequency; in turn, frequency was positively and robustly associated with months per year and minutes per week.

Lastly, Table 2 shows that autonomy need satisfaction was positively and significantly related with all regularity characteristics including minutes per week but not minutes per day and that relatedness need satisfaction was positively related with minutes per day and per week devoted specifically to the main PA.

Fusion coefficients resulting from the hierarchical clustering (Ward’s method) suggested a four-cluster solution (Table 3), and a subsequent iterative step, using final cluster centers from the hierarchical step as initial centers for the iterative step, required only three iterations to reach the stopping criterion (changes in cluster centers < 2% of initial minimum distances). Nonetheless, we tested three-, four-, and five-cluster solutions. The four-cluster solution was preferred over the five-cluster solution, given its greater stability between hierarchical and iterative steps, as judged by its strong Cohen’s Kappa of 0.80 (vs. Cohen’s Kappa five-cluster = 0.76). Also, the four-cluster solution was preferred over the three-cluster solution (Cohen’s Kappa = 0.84) given its greater explanation of variance in clustering variables (enjoyment = 37 vs. 28%; competence = 45 vs. 36%; social = 63 vs. 62%; fitness = 50 vs. 43%; and appearance = 56 vs. 36%). The five motives contributed significantly to distinguishing the emerging clusters, but the social motive, followed by the appearance motive, was the most relevant variable for cluster distinction (see Table 4).

**TABLE 3:** Fusion coefficients for PA motives.

Group number	Fusion coefficient
1	3,850.38
2	2,830.03
3	2,244.61
4	1,932.60
5	1,728.63
6	1,575.19
7	1,467.56
8	1,360.46
9	1,285.82
10	1,223.56

**TABLE 4:** PA motives, characteristics of practice, and psychological need satisfaction by cluster.

	Extrinsic-motives (*n* = 103)	All-motives (*n* = 140)	Intrinsic-motives (*n* = 119)	Low-motives (*n* = 61)	*F*	*p*

	***M* (*SD*)**	***M* (*SD*)**	***M* (*SD*)**	***M* (*SD*)**		
Enjoyment motive	5.13 (1.37)	6.42 (0.62)	6.42 (0.60)	5.13 (1.23)	61.73	<0.001
Competence motive	5.25 (1.21)	6.33 (0.67)	5.89 (0.79)	4.04 (0.99)	100.37	<0.001
Social motive	2. 44 (1.15)	5.57 (0.93)	5.69 (0.95)	4.00 (1.26)	238.07	<0.001
Fitness motive	6.19 (0.66)	6.56 (0.50)	5.70 (0.80)	4.13 (1.05)	168.62	<0.001
Appearance motive	5.46 (1.13)	5.92 (0.66)	3.63 (1.01)	2. 95 (1.26)	201.85	<0.001
Months per year PA1	9.9 (2.7)	10.1 (2.6)	9.2 (3.2)	9.2 (3.2)	2.508	0.058
Frequency PA1	5.7 (0.6)	5.8 (0.7)	5.5 (1.0)	5.1 (1.4)	8.547	<0.001
Days per week PA1	3.3 (1.4)	3.2 (1.3)	3.3 (1.5)	3.4 (1.4)	0.600	0.614
Minutes per day PA1	78 (40)	102 (54)	136 (83)	129 (125)	13.060	<0.001
Minutes per week PA1	212 (191)	280 (214)	320 (365)	275 (483)	3.320	0.020
Total number of PAs	1.4 (0.8)	1.6 (1.3)	1.5 (1.1)	1.5 (0.9)	0.797	0.496
Total minutes per week	256 (266)	357 (346)	379 (412)	360 (562)	2.646	0.049
PA1 competitive (%)	20	41	57	40	14.905	<0.001
PA1 with team (%)	19	56	79	42	35.132	<0.001
PA1 with coach (%)	28	52	69	48	14.448	<0.001
PA1 league (%)	7	25	45	38	19.994	<0.001
PA1 federation (%)	8	27	43	35	13.420	<0.001
WHO recommendation (%)	63	76	71	53	4.100	0.007
Competence NS	4.74 (0.75)	5.11 (0.77)	4.86 (0.83)	4.19 (1.07)	11.983	<0.001
Autonomy NS	4.84 (0.92)	4.83 (0.94)	4.28 (1.07)	4.24 (1.06)	12.796	<0.001
Relatedness NS	3.14 (1.28)	4.41 (0.97)	4.75 (0.70)	3.78 (1.05)	56.890	<0.001

*PA = physical activity, PA1 = most regular specific physical activity practiced. NS = need satisfaction. The following characteristics (competitive, with team, with coach, league, and federation) were coded as 1 if participant opted in or 0 if they did not. Frequency was coded as follows: at least once weekly (=6), at least once every 2 weeks (=5), at least once monthly (=4), at least once every 3 months (=3), at least once every semester (=2), and at least once per year (=1).*

**FIGURE 1:**
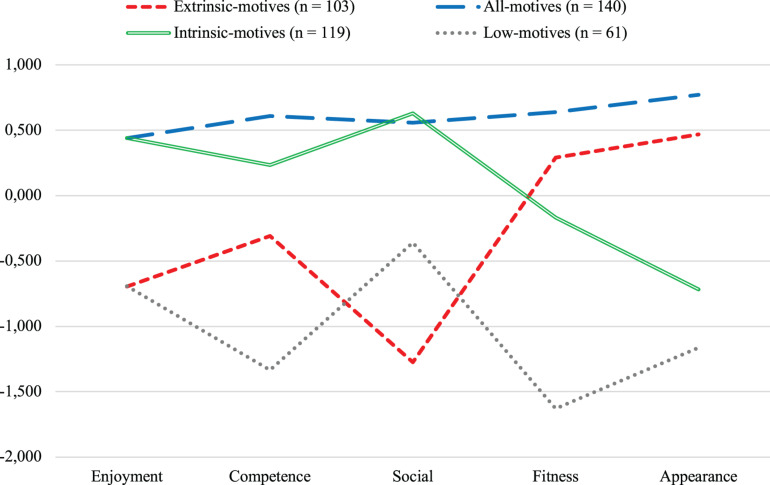
Four profiles of motives for physical activity (*Z* values).

Based on stable distinct naturally occurring combinations of the five motives for PA, the following four clusters emerged *via* hierarchical followed by iterative cluster analysis (Figure 1). Emerging clusters were as follows: one *extrinsic-motives* cluster (*n* = 103), with fitness and appearance motives above the mean; one *all-motives* cluster (*n* = 140), with all five motives above the mean; one *intrinsic-motives* cluster (*n* = 119), with all three intrinsic motives (enjoyment, competence, social) above the mean; and one *low-motives* cluster (*n* = 61), with all motives below the mean. Gender and age did not significantly differ between clusters. *Post hoc* tests (Dunnett’s *t*) to compare cluster centers one-to-one revealed significant (*p* < 0.05) differences in all pairs, except for intrinsic-motives and all-motives clusters, tied for the highest rank in both enjoyment and social motives; and extrinsic-motives and low-motives clusters, tied for the lowest rank in enjoyment.

As reported in Table 4, practice regularity measures were significantly different between groups. As regards months per year and frequency of PA, members of the extrinsic-motives (9.85) and all-motives clusters (10.16) practiced—in average—10 months per year and nearly once per week (respectively, 5.73 and 5.78, on a six-point Likert scale with 6 = “at least weekly PA”). Members of the intrinsic-motives and low-motives clusters practiced slightly above 9 months per year (respectively, 9.33 and 9.26); out the latter two, intrinsic-motives cluster members averaged = 5.5 in PA frequency (between weekly = 6 and biweekly = 5), whereas low-motives cluster members scored only slightly above (=5.13) the biweekly mark of 5.

Significant between-group differences were also reported in duration of single main PA sessions. For extrinsic-motives cluster members, duration was the shortest (79 min), relative to other clusters, whereas for intrinsic-motives and low-motives cluster members, it was the longest (respectively, 133 and 134 min). All-motives cluster members typically practiced 100 min per session. Similarly, significant between-group differences were reported in minutes per week for main PA and total weekly PA time, but not in the number of days per week devoted to the main PA (slightly above 3 days per week) nor number of total PAs practiced (between one and two physical activities) across all clusters (Table 4).

Among members of the low-motives cluster, only 52.5% complied with the WHO’s recommendations of 150 min of PA per week; among extrinsic-motive, all-motives, and intrinsic-motives cluster members, percentages of compliance were, respectively, 63.1, 75.7, and 70.6%.

**FIGURE 2:**
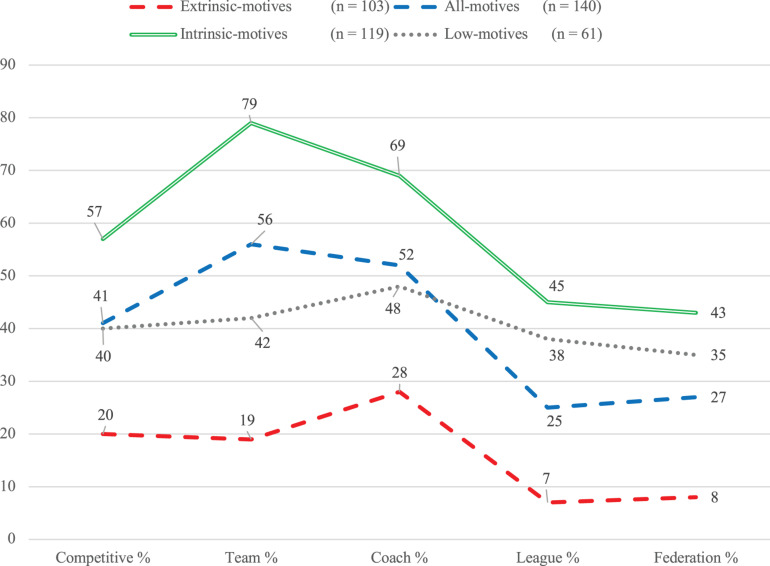
Percentages of characteristics of physical activity by motive profile.

Furthermore, significant between-group differences were observed in the percentages of members who practiced competitively, as a team, with a coach, in a league, or as part of a federation (Table 4). Members of the intrinsic-motives cluster reported the highest percentages in all five aforementioned aspects, whereas extrinsic-motives cluster members reported the lowest. All-motives cluster members ranked the second highest in the percentage of members practicing competitively, as a team, and with a coach, and interestingly, low-motives cluster members ranked the second highest in the percentage of practice in leagues and federations (see Figure 2).

As can be observed in Table 5, practically half of the members of the extrinsic-motives and the all-motives clusters practiced only on weekdays and one-fourth of them practiced both on weekends and weekdays; smaller percentages practiced only on weekends or without a specific set day. Contrarily, among members of the low-motives and intrinsic-motives clusters, close to one in every five practiced only on weekends and, respectively, around 10 and 15% practiced without a specific set day.

**TABLE 5:** Type of day devoted to PA by motive profile.

Type of day devoted to PA	Total sample (*N* = 423)		Extrinsic-motives (*n* = 103)	All-motives (*n* = 140)	Intrinsic-motives (*n* = 119)	Low-motives (*n* = 61)	χ^2^	*p*
	*n*	%						
Only on weekdays	175	41.4	52.4	50.7	26.9	29.5	24.056	<0.001
Only on weekends	59	13.9	11.7	10.0	18.5	18.0	5.162	0.160
Both weekends and weekdays	149	35.2	25.2	33.6	45.4	36.1	10.061	0.018
No regular type of day	40	9.5	10.7	5.7	9.2	16.4	5.904	0.116

**FIGURE 3:**
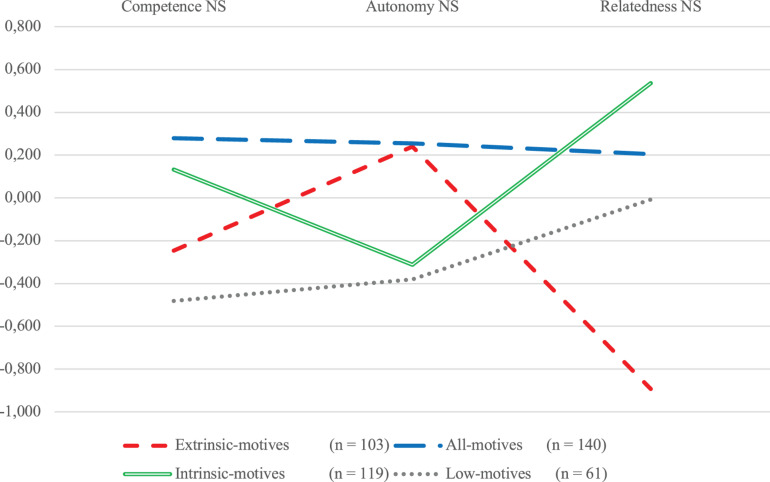
Psychological need satisfaction in PA by motive profile (*Z* values).

Lastly, Figure 3 shows the levels of PNS in PA by cluster. Extrinsic-motives cluster members reported above-average autonomy need satisfaction, but below-average competence need satisfaction and the lowest relative relatedness need satisfaction. Conversely, intrinsic-motives cluster members reported the highest relative relatedness need satisfaction, average competence need satisfaction, and below-average autonomy need satisfaction. All-motives cluster members were the only participants to report above-average satisfaction of all three psychological needs in PA, whereas low-motives cluster members reported the lowest levels of competence need satisfaction relative to all the three other clusters. *Post hoc* tests (Bonferroni for autonomy and competence need satisfaction and Dunnett’s *t* for relatedness need satisfaction in PA) revealed that one-to-one between-cluster differences in need satisfaction were significant, except for those in competence need satisfaction, between, respectively, extrinsic-motives and low-motives clusters and between intrinsic-motives and all-motives clusters; those in autonomy need satisfaction, respectively, between intrinsic-motives and low-motives cluster members and between all-motives and extrinsic-motives cluster members; and those in relatedness need satisfaction between all-motives and low-motives cluster members.

## Discussion

The present work has analyzed the motive base of university students in PA and the relationships between motives, characteristics of practice regularity, and basic psychological need satisfaction, with the goal of contributing relevant evidence useful for the understanding and promotion of university students’ health habits in PA. As described in the following sections, the present findings support the relationship between PNS and motives (in this case for PA) (Ryan and Deci, 2000; Milyavskaya and Koestner, 2011; Battistelli et al., 2016). To this regard, we identify distinct motive profiles, which replicate those found in previous studies (Nuviala Nuviala et al., 2013) and confirm that these diverse motive profiles indeed are associated with different levels of PNS, thus, in a broad sense, providing evidence for SDT assumptions regarding the association between PNS and motivation (Sebire et al., 2008; Ryan and Deci, 2017). All in all, our study extends the evidence supporting the existence of distinct profiles of motives for physical activity, by identifying and describing four distinct naturally occurring groupings, specifically, among university students. Furthermore, emerging clusters are compared regarding the regularity of their practice and their derived levels of psychological need satisfaction, in order to help PA researchers and educators in understanding and supporting their subjects’/students’/coaches’ complex motive base in simpler and tailored ways.

### From the Four Motive-Based Clusters to the Time Invested in PA

The retained four-cluster solution was stable between hierarchical and iterative steps, it required few iterations to stabilize, and it explained considerable proportions of variance in clustering variables. Furthermore, three out of the four identified clusters (e.g., all-motives, intrinsic-motives, extrinsic-motives) are practically identical to three clusters found in previous studies in the same country with the same version of the MPAM-R (Nuviala Nuviala et al., 2013), which also emerged with scores around-and-above average, respectively, for all-motives, for extrinsic-motives (fitness and appearance), and for intrinsic-motives (enjoyment, competence, social). Nonetheless, the additional cluster that emerged in the present study (low-motives) is theoretically reasonable, given that behavior can originate in internal motives (needs, cognitions, emotions) and in external events; thus, it is theoretically plausible (Reeve, 2009) that some participants may have practiced PA for cognitions or emotions not considered in the MPAM-R, or even compelled by external events and not by internal motives. These findings also dialogue with previous latent profile analysis showing that two out of the five motivational profiles identified in physical education scored above-average only in external regulation (Wang et al., 2016).

Members of clusters with different motive base also reported different PA time investment and characteristics. Firstly, in line with SDT, members of intrinsic-motives and extrinsic-motives clusters reported, respectively, the highest and the lowest (379 vs. 256 min) average weekly PA practice, whereas members of all-motives and low-motives clusters scored similar in total weekly PA practice (357 vs. 360 min). Furthermore, the proportions of students who reached the WHO’s weekly PA practice time recommendation (150 min) were also different when comparing between members of different clusters: four out of five (all-motives), two out of three (extrinsic motives), three out of five (intrinsic-motives), and only one out of two (low-motives). These findings extend the evidence supporting the notion that types of motives for PA are related with regularity of time investment in PA. Regarding PA regularity characteristics, relative to the rest of the participants, intrinsic-motives cluster members reported the highest proportions of practicing PA competitively, in teams, with coach, in leagues, and in federations and the highest proportions of practice reported during only weekends and during both weekdays and weekends. This finding possibly attests to the fact that competitive team sports require thorough coordination for participants to find common available time slots for group practice.

### What Is Obtained and Why When Practicing Physical Activity

The fact that intrinsic-motive cluster members scored relatively higher in the enjoyment and social motives then in the competence motive suggests that, as has been theorized, perceived competence serves the purpose of promoting enjoyment and, thus, intrinsic motivation (Deci, 1975), rather than the other way round. In the case of strictly intrinsically motived PA practice, enjoyment could be thought of as an end, and competence could be seen as the means to it. Arguably, the competence motive could be thought of as partly extrinsic (even though, of course, with an intrinsic value), given that the natural outcome of expressing competence (hence, the motivator) could be competence-derived enjoyment. On the other hand, a true competence motive, where being competent itself was the motivator, would depend on a cognitive identification with the goal of enhancing or expressing high competence. This nuance in the relationship between competence and enjoyment could provide an interpretive context for the statistically significant difference in competence motive levels between intrinsic-motives and all-motives cluster members: the latter (regardless of being more extrinsically motived and less competitive) carrying more competence-striving cognitions, but not necessarily being more competent or enjoying more than intrinsic-motives cluster members.

Furthermore, interestingly, both clusters including above-average extrinsic motives (e.g., all-motives and extrinsic-motives clusters) reported the greatest proportions of regular physical activity during weekdays. These findings suggest that the extrinsic value ascribed to PA may be critical for people to consider weekday practice as important as to separate specific time for it from competing typically extrinsic goals. Contrastingly, intrinsic-motives cluster members reported the smallest proportion of students participating only on weekdays, suggesting that PA characteristics integrated more with weekend leisure time than with regular weekday routine and habit. Future longitudinal studies are needed to discern if the fact that PA recedes during higher education is related with weekday-versus-weekend time use for PA (hints are that intrinsic-motives cluster members would be at a greater risk of PA slipping out of their healthy lifestyle during higher education relative to students with extrinsic motives—or with a combination of intrinsic and extrinsic motives), given the high demand of studies on weekday time investment.

Lastly, the four identified clusters also showed different levels of PNS. It has been established that PNS is positively associated with autonomous motivation (Milyavskaya and Koestner, 2011), which suggests that mainly intrinsically motived PA could depend on PNS. Nonetheless, the present person-centered approach allows for nuanced observations and interpretations regarding PNS in PA practice, depending on specific naturally occurring combinations of motives.

### Psychological Need Satisfaction in Practicing PA

The cluster with the highest relative intrinsic motivation (intrinsic-motives) not necessarily always yields the highest, broadest, or more balanced PNS in PA. In fact, seemingly, the competitive and team-based structure of PA among intrinsic-motives cluster members (mainly motived by enjoyment and social relatedness) allows for a high level of relatedness need satisfaction and a slightly above-average competence need satisfaction, but limits the satisfaction of the need for autonomy (to levels similar to those of the low-motives group). This finding may in part reflect the operationalization of autonomy implied in the used measure, in which participants reported how much decision they had about how to exercise in their reported PA. In other words, participants of league-competitive team-based sports may logically have less possibility to decide about how to practice and could thus have reported lesser autonomy need satisfaction than independent individual practitioners who could decide how to practice. It is not safe to assume that intrinsic-motives cluster members feel autonomy-thwarted just for not being able to change the rules of the sport they practice. Furthermore, the original scale already recommended caution given that the autonomy subscale captured only a portion of the construct’s content (Wilson et al., 2006).

Extrinsic-motives cluster members reported high levels of autonomy need satisfaction, possibly given the fact that their practice was the least coached, competitive, and team-league-or-federation-based of the four clusters. This does not make the extrinsic-motives cluster members necessarily the most autonomous practitioners of the four groups, but specifically reflects their control over their concrete practice behavior, the trade-off being not practicing with others (which would enable relatedness need satisfaction), nor having specific criteria for assessing and socially comparing performance (which would enable competence need satisfaction), two aspects that may be perceived as irrelevant by extrinsically motived PA practitioners.

The all-motives cluster had the most robust and balanced PNS across the three needs. From this perspective, it is reasonable that this cluster also has the greatest number of participants reaching the WHO recommendations of weekly PA. The fact that their PA practice satisfies their three psychological needs in a balanced manner should be a strong base for their intrinsic motives to remain in place, but, additionally, having cognitions and emotions regarding fitness and appearance (as non-need-based motivators) may promote the allocation of weekday time for PA practice, rendering this group the one with the greatest regularity of practice at the same time with the highest autonomy need satisfaction.

Conversely, low-motives cluster members reported both below-average need satisfaction in all three needs and below-average levels in all five motives, which leads to two possible interpretations: either these students practice PA due to external regulation based on external events or, alternatively, they are amotivated by PA and find themselves in a transition to another type of practice or to ceasing to practice all in all. The second interpretation finds some ground in observing that, regarding scores for practice in leagues and federations, low-motives cluster members’ PA practice is similar to the intrinsic-motives cluster, which (given their low motives and need satisfaction) could be due to the external regulation for practice implied in practicing PA with coach, or in teams, contests, leagues, or federations.

### Final Remarks

The relevance and robustness of these findings do not mean that the study was conducted without limitations. Particularly, we used an intentional sampling method aimed at yielding participants from diverse fields of study who practiced physical activity. To this respect, it is relevant to mention that the specific characteristics of these profiles may set conditions for the fulfilment of other activities, obligations, or other aspects of day-to-day life, especially during weekdays (in which a majority of students practiced PA). In any case, the detailed description of profiles relative to motive combinations gives researchers and practitioners the possibility to analyze and implement tailored interventions aimed at promoting PA, considering its short- and long-term effects on health. Concretely, programs could be aimed at promoting higher intrinsic motivation levels through tailored activities. About such interventions, it has been argued that, on the one hand, the accumulated knowledge about motivation in PA requires more examples, systematicity, and longitudinal approaches (Belogianni and Baldwin, 2019; Huffman et al., 2020), and on the other hand, there have been reports of positive effects of the incorporation of new technologies in increasing PA in the youth (Wibowo et al., 2020). These evidences highlight that, looking into the future, a joint venture between longitudinal research designs and more highly sensitive measurement devices, aligned with day-to-day lives of the youth, will be important for the understanding of young people’s motives for physical activity.

## Data Availability Statement

The datasets analyzed for this study can be found in the Dipòsit Digital (digital archives) from the University of Barcelona (http://diposit.ub.edu/dspace/handle/2445/171472).

## Ethics Statement

All subjects gave written informed consent prior to the collection of the research data. The ethical requirements of the University of Barcelona Bioethics Commission (CBUB, Institutional Review Board IRB00003099) were applied to the current study, which meant that additional approval for the research was not required because the data obtained did not involve animal or clinical experimentation. Additionally, this study complies with the recommendations of the General Council of Spanish Psychological Associations (Consejo General de Colegios de Psicólogos), the Spanish Organic Law on Data Protection (Jefatura del Estado, 1999), and the Declaration of Helsinki (World Medical Association, 2013).

## Author Contributions

RV is the author who conceived and designed this manuscript. He was also responsible for drafting the whole work and revising it critically for important intellectual content. NC was mainly responsible for the research that gave the data gathered and for revising the manuscript critically for important intellectual—theoretical and methodological—content. JP was responsible for revising the manuscript critically for important intellectual—theoretical and methodological—content. All authors contributed to the article and approved the submitted version.

## Conflict of Interest

The authors declare that the research was conducted in the absence of any commercial or financial relationships that could be construed as a potential conflict of interest.
